# Technical aspects and interpretation of oscillometry

**DOI:** 10.36416/1806-3756/e20250163

**Published:** 2025-09-22

**Authors:** Irlanda Alvarado-Amador, Mario Arturo Flores-Valadez, Laura Gochicoa-Rangel

**Affiliations:** 1. Instituto Nacional de Enfermedades Respiratorias - INER - Ismael Cosío Villegas, Ciudad de Mexico, Mexico.

Oscillometry is a noninvasive pulmonary function test that measures respiratory system impedance during tidal breathing. It does not require forced maneuvers, and by using a low-amplitude oscillatory signal, it assesses respiratory resistance and reactance.^1^

Resistance reflects opposition to airflow. Low-frequency measurements (e.g., resistance at 5 Hz-R5-and resistance at 6 Hz-R6) represent total airway resistance, whereas high-frequency measurements (e.g., resistance at 19 Hz-R19-and resistance at 20 Hz-R20) reflect central airway resistance. The difference between low and high frequencies (e.g., R5-R20) offers insight into distal airway involvement. Reactance encompasses two components: elastance, which quantifies the pressure required to overcome the resistance of the lung to volume change, thereby reflecting its stiffness or decreased compliance; and inertance, which mainly reflects pressure losses from gas acceleration in the central airways. More negative values of reactance at 5 Hz (X5) indicate increased elastance. The resonant frequency (Fres), where total reactance equals zero, increases with decreased lung compliance. The area of reactance (AX), which is defined as the area under the X5-Fres curve, quantifies the reactive load of the respiratory system and is a sensitive indicator of peripheral airway obstruction.^2^

Reference equations developed with the same device must be used in order to ensure accurate interpretation because results are not interchangeable between devices. It is also important to confirm that the equation covers the age range of the study population in order to avoid misclassification of patterns (Table 1). 

**Table 1:** Oscillometric patterns of lung function.

Functional pattern	R5	R20	R5-R20	X5	AX	Fres
Normal	Normal	Normal	Normal	Normal or less negative	Normal	Normal
Peripheral obstruction	Normal or increased	Normal or increased	Increased	Normal or Increased (more negative)	Increased	Normal or increased
Central obstruction	Increased	Increased	Normal	Normal	Normal	Normal
Probable restriction	Normal	Normal	Normal	Increased (more negative)	Increased	Normal or increased

ULN: upper limit of normal; LLN: lower limit of normal; R5: resistance at 5 Hz; R20: resistance at 20 Hz; R5-R20: difference between R5 and R20; X5: reactance at 5 Hz; AX: area of reactance; and Fres: resonant frequency. Resistances, AX and Fres are considered normal if the value is ≤ULN. Reactances are considered normal if the value is ≥LLN.

Oscillometry has shown clinical value across several respiratory conditions. In asthma, it can detect small airway dysfunction even when FEV_1_ is normal. Abnormal R5-R20, X5, and AX values are common and correlate with disease control. In bronchopulmonary dysplasia, preterm individuals often present with elevated resistance and AX, as well as more negative reactance values, suggesting persistent mechanical impairment. In neuromuscular disorders, increased R5, Fres, and AX values, as well as more negative reactance values, reflect a restrictive pattern caused by reduced lung volumes and increased elastic load from respiratory muscle weakness.^3^

In acute respiratory failure, AX is often the most affected parameter, indicating elevated elastance and heterogeneous tissue mechanics. Reactance becomes more negative, reflecting increased lung stiffness, whereas resistance may remain within normal limits. These values typically improve with recovery, making oscillometry a useful tool for monitoring disease progression and therapeutic response.^4^

Regarding bronchodilator response, King et al.^2^ proposed the following thresholds: ≥ 40% reduction in low-frequency resistance (R5 and resistance at 6 Hz); a ≥ 50% increase in X5; or a ≥ 80% decrease in AX. Bickel et al.^1^ suggested more conservative cutoffs based on ROC analysis: a ≥ 30-35% reduction in R5; a ≥ 8.6% reduction in resistance at 10 Hz; or ≥ 29.1% reduction in AX. 

Although no specific oscillometry phenotype for restrictive lung disease has been defined, findings in a veteran cohort suggest that more negative values of X5, an increased AX, and a higher Fres are associated with a restrictive pattern, likely reflecting increased lung stiffness.^5^

In conclusion, oscillometry is a sensitive and versatile technique that complements spirometry in a wide range of clinical contexts. However, further standardization-particularly in the interpretation of parameters associated with restrictive patterns-is required to enhance its diagnostic accuracy and clinical integration. 
